# The Medical Association of Georgia

**Published:** 1889-05

**Authors:** 


### THE MEDICAL ASSOCIATION OF GEORGIA.

The annual meeting of the Medical Association of Georgia held in Macon last month was a signal success from a social point of view. The proverbial hospitality of the Central City was thoroughly exemplified in the handsome entertainment of the physicians from abroad who were present at the meeting. We are confident that every member carried with him to his home pleasant memories of the occasion, which will linger about him like a fragrant aroma for many days to come. Not only the profession of Macon, but her citizens as well, including her charming women, contributed to render the social aspect of the meeting all that could be desired.

It is unfortunate that as much cannot be said of the meeting from the scientific side of the question. Of a very respectable number of papers which had been prepared for the occasion, only a few found opportunity to be read, and the remainder are doomed to be buried in the Transactions, where they will benefit neither their authors nor the profession at large. The reason for this unfortunate condition of things is found in the fact that more than a half of the time of the Association was frittered away in tiresome consideration and discussion of business matters, all of which should have been disposed of in a single session of a half a day. The main object of a medical association is scientific work, and it is this feature which alone can induce busy practitioners to sacrifice time and money in attendance upon medical meetings. The paucity of such work at the last few meetings of the Association is an obvious explanation of the small attendance at Macon, the most accessible point in Georgia, and the conspicuous absence of all but a few of the men who are looked upon as leaders of the profession in this State. He is no pessimist who predicts that a continuance of this state of things will ultimately result in the death of the Association, or a condition of uselessness which will be worse than death.

And yet the meeting was not without its redeeming features.

The masterly address of the President was a marvel of wide research and original thought, and formed a contribution of permanent value to medical literature. Other papers which were read showed the results of original research and wide experience, and were listened to with marked attention and interest.

Two important changes were made at this meeting in the policy of the Association in regard to the publication of papers. The old rule was abolished which provides that no paper shall appear in the Transactions which has been published elsewhere. Heretofore this rule has resulted in the exclusion from the Transactions of the best papers read at the meetings, since no writer will consent to spend time in the preparation of a valuable paper, for the exclusive benefit of the small circle of readers reached by the Transactions. The best writers in the Association have preferred to reach the profession at large by the publication of their papers in the medical journals. The Transactions have therefore failed to furnish a fair presentation of the best work of the Association.

The second change of policy consists in the adoption of the rule that no paper shall be read before the Association until a complete copy shall have been placed in the hands of the Secretary. By this plan a speedy issue of the Transactions will be secured, and authors will not be compelled to wait eight or ten months before their productions will appear in print.

The recent meeting demonstrated that there is abundant material in the Association for good scientific work. It also demonstrated that under the present arrangement there is not sufficient stimulus and encouragement for such work. The remedy for this state of things is to be found only in a systematic pre-arranged programme for each meeting, which shall make the business portion of the meeting secondary to the reading and discussion of papers. The programme committee failed to accomplish this end at the last meeting on account of the failure of members to send in the titles of their papers beforehand, as required by a resolution adopted in Rome a year ago. We learn that this failure was due to a misunderstanding of the rules under which the committee was working. We trust that before the next meeting every member will so familiarize himself with the rules as to co-operate with the programme committee in obviating the objectionable features which were so conspicuously present in Macon.

